# Glycans and Glycan-Binding Proteins as Regulators and Potential Targets in Leukocyte Recruitment

**DOI:** 10.3389/fcell.2021.624082

**Published:** 2021-02-04

**Authors:** Franziska Krautter, Asif J. Iqbal

**Affiliations:** Institute of Cardiovascular Sciences, University of Birmingham, Birmingham, United Kingdom

**Keywords:** glycan, glycan-binding protein, leukocyte recruitment, transmigration, lectins, glycomimetics

## Abstract

Leukocyte recruitment is a highly controlled cascade of interactions between proteins expressed by the endothelium and circulating leukocytes. The involvement of glycans and glycan-binding proteins in the leukocyte recruitment cascade has been well-characterised. However, our understanding of these interactions and their regulation has expanded substantially in recent years to include novel lectins and regulatory pathways. In this review, we discuss the role of glycans and glycan-binding proteins, mediating the interactions between endothelium and leukocytes both directly and indirectly. We also highlight recent findings of key enzymes involved in glycosylation which affect leukocyte recruitment. Finally, we investigate the potential of glycans and glycan binding proteins as therapeutic targets to modulate leukocyte recruitment and transmigration in inflammation.

## Introduction

**Figure 1:**
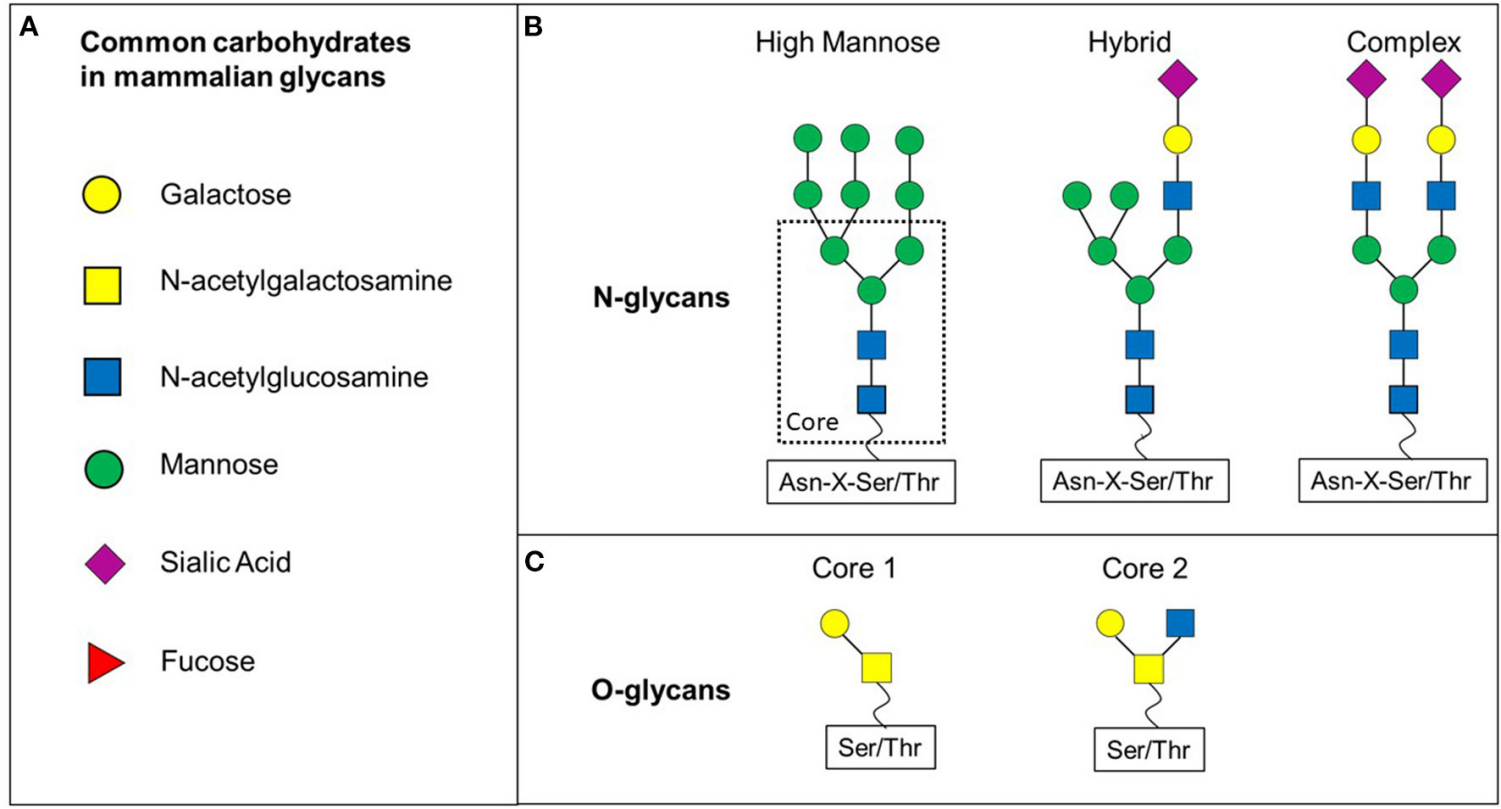
N- and O-glycan differ in their core structure. **(A)** Various carbohydrates are making up mammalian glycans. **(B)** N-glycans share a common core structure. The carbohydrates added to the core structure define the three subtypes of N-glycans: high-mannose, hybrid and complex. **(C)** O-glycans discussed in this review are based on core 1 or core 2 O-glycans.

Glycosylation is a post-translational modification whereby carbohydrates are added to proteins or lipids to expand their functional profile. Approximately 10% of the human genome encodes for proteins which play roles in glycosylation and about 10^12^ possible glycan structures have been previously predicted, highlighting the importance and complexity of this post-translational modification (Laine, 1994; Haslam et al., 2008). The majority of glycan structures are found on the cell surface, but can also be detected intracellularly in the cytoplasm and nucleus. Glycosylation is a tightly controlled process involving glycosyltransferases and glycosidases which form the carbohydrate structures dependent upon sugar precursors, cellular environment and cell type (Reily et al., 2019). Structurally and biosynthetically, glycans can be divided into N- and O-glycans (Figure 1).

N-glycans all share the same core structure and are added to an asparagine (Asn) side chain via N-linked glycosylation initiated by oligosaccharyltransferase complex in the endoplasmic reticulum membrane (Schjoldager et al., 2020). N-glycans are attached to Asn located in a sequence of Asn-X-Serine/Threonine, whereby X can be any amino acid apart from proline (Figure 1B). The N-glycan core structure comprises of two N-Acetyl-D-glucosamine (GlcNAc) and three mannose molecules. This core structure is expanded through galactosylation, further GlcNAclyation, sialylation or fucosylation. These additions define three subclasses of N-glycans: high-mannose, hybrid and complex (Figure 1B).

O-glycans have eight different core structures and are attached to an -OH group of either serine or threonine via a O-glycosidic bond with an N-Acetyl-D-galactosamine (GalNAc) (Figure 1C) (Brockhausen et al., 2009). The glycan is attached to the amino acid residue via one of at least 21 known polypeptide-N-acetylgalactosaminetransferases (ppGalNAcT-1 to−21) (Brockhausen et al., 2009). However, further glycosyltransferases are involved in the formation of O-glycans by adding to specific core structures (Figure 1C). Not only the availability of the enzyme substrate, but also the subcellular localisation affects the activity of these enzymes and therefore contributes to a wide range of (branched) O-glycans (Brockhausen et al., 2009).

Due to the great heterogeneity of structures, the functions of glycans also vary greatly. The biological roles of mammalian glycans can be broadly classified into three groups: (I) structural and modulatory functions such as in membrane organisation and epigenetic histone modifications(II) extrinsic recognition, for example of bacterial or viral adhesins (III) intrinsic recognition, for example in cell adhesion or intercellular signalling (Varki, 2017). We will focus on the role of glycans in leukocyte recruitment and migration in this review. This function, as any other, requires the glycomic code to be translated into function. Glycan-binding proteins are proteins which recognise and bind specific sequences of glycans and therefore facilitate cellular processes based on the glycomic profile. Various families of these proteins have been described: -galactoside binding lectins (Galectins, Gal), C-type lectins which require calcium for binding, I-type lectins which are a subset of the immunoglobulin superfamily, L-type lectins which are similar to leguminous plant lectins, P-type lectins which recognise phosphorylated mannose residues and R-type lectins which have a similar carbohydrate recognition domain (CRD) as ricin (Varki et al., 2009). However, not all of the mentioned lectin families have been shown to play a role in leukocyte migration.

**Figure 2:**
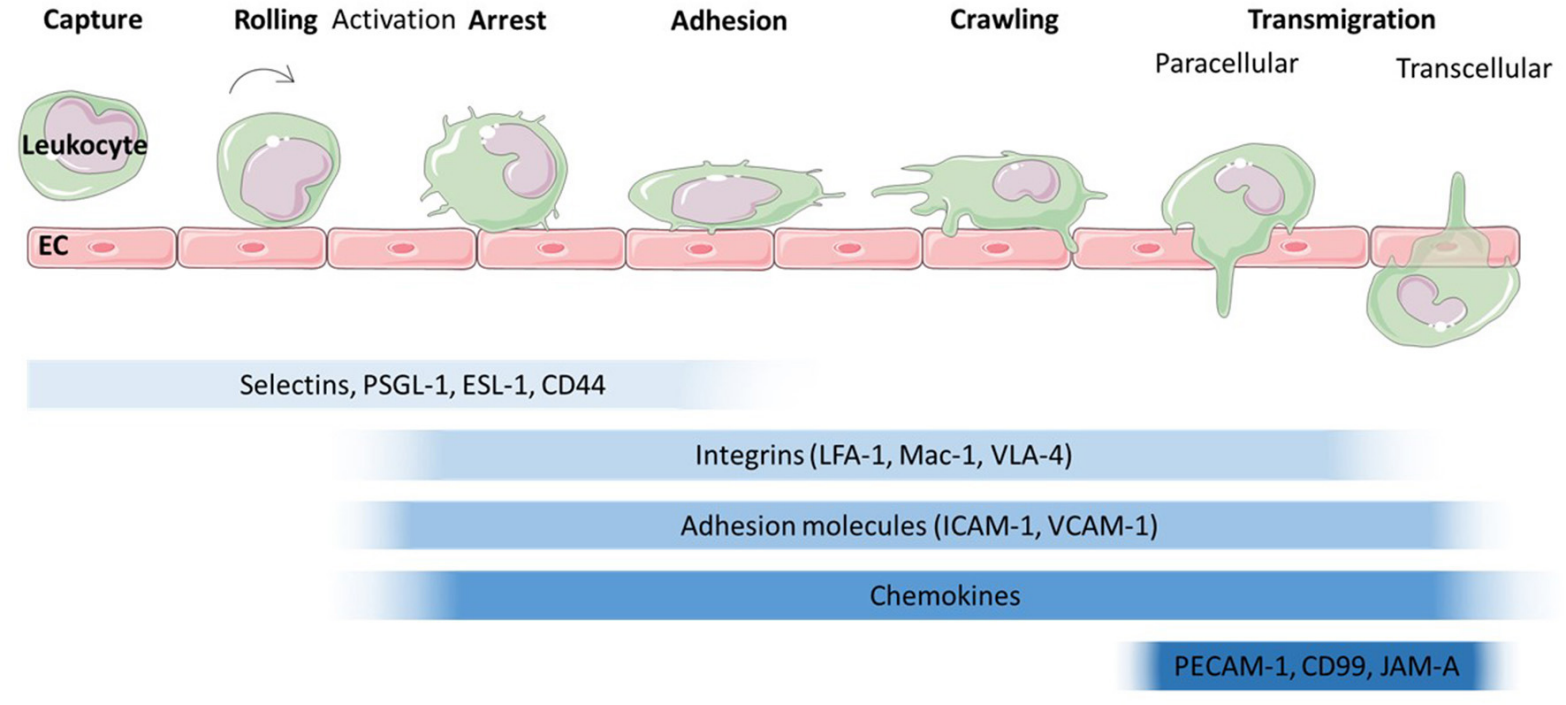
Leukocyte migration cascade across endothelium. The leukocyte migration cascade comprises of a sequence of steps which are mediated by a range of proteins. The initial steps of capture and rolling are dominated by the interaction of selectins with proteins such as P-selectin glycoprotein ligand (PSGL)-1, E-selectin ligand (ESL)-1 and Cluster of differentiation (CD) 44. Activation leads to a conformational change in integrins such as lymphocyte function-associated antigen (LFA)-1, Macrophage (Mac)-1 antigen and Very Late Antigen (VLA)-4 which then interact with adhesion molecules such as Intercellular Cell Adhesion Molecule (ICAM-1) and Vascular Cell Adhesion Molecule (VCAM)-1. Together with chemokines presented on the endothelial surface, arrest and adhesion are triggered. The final step, transmigration through the endothelial cell layer is mediated by proteins such as platelet endothelial cell adhesion molecule (PECAM)−1, CD99 and junctional adhesion molecule (JAM) -A.

The migration of leukocytes was first described in the nineteenth century (Dutrochet, 1824; Wagner, 1839) and has since been characterised extensively. Leukocyte recruitment to sites of inflammation, infection or tissue damage involves a series of tightly regulated, co-ordinated steps (Figure 2). Traditionally, the leukocyte recruitment cascade was described as a three-step process of rolling, activation and firm adhesion. However, this model has been expanded upon to include slow rolling, crawling and transmigration (Ley et al., 2007). Numerous proteins involved in the regulation of these steps have been identified over the years (Figure 2) [we refer to the excellent review by Ley et al. (2007)]. Briefly, leukocytes are captured and roll along activated endothelium through interactions between selectins (E-, L-, and P-selectin) and glycosylated proteins such as P-selectin glycoprotein ligand (PSGL)-1, CD44 and E-selectin ligand (ESL)-1 (Katayama et al., 2005; Hidalgo et al., 2007). This leads to activation of leukocytes and conformational changes to integrins, enabling integrin-mediated leukocyte rolling followed by irreversible binding between integrins, such as Macrophage (Mac)-1 antigen found on neutrophils and monocytes, lymphocyte function-associated antigen (LFA)-1, found on lymphocytes, monocytes and neutrophils or Very late antigen (VLA)-1 found on monocytes and T-lymphocytes and adhesion molecules such as intercellular adhesion molecule (ICAM)-1 and vascular cell adhesion molecule (VCAM)-1 on the endothelium (Diamond and Springer, 1993; Mitroulis et al., 2015). Which types of integrins and adhesion molecules dominate in the adhesion is cell type and tissue dependent (Rossaint and Zarbock, 2013; Maas et al., 2018; Schnoor et al., 2021). For example, integrin-mediated lymphocyte and monocyte rolling is mainly dependent on VLA-1 (Berlin et al., 1995; Huo et al., 2000; Chan et al., 2001; Singbartl et al., 2001). ß2-integrins such as LFA-1 which interacts with ICAM-1 on the endothelial surface also support rolling, as has been shown for mouse neutrophils (Kadono et al., 2002). Integrin-mediated adhesion is also regulated by chemokines which trigger integrin activation and crawling. However, more recent studies have also highlighted tissue specific differences in leukocyte recruitment independent of selectins and integrins [reviewed in Schnoor et al. (2021)]. For example dipeptidase-1 acts as adhesion molecule for murine neutrophils in lung and liver, but not in the cremaster muscle (Choudhury et al., 2019). Various studies have identified ICAM-1, VCAM-1, platelet endothelial cell adhesion molecule (PECAM)-1, junctional adhesion molecule (JAM)-A and -C as well as ICAM-2 and Cluster of Differentiation (CD)99 as key molecules involved in transmigration, whereby leukocytes cross the endothelial layer in a trans- or paracellular manner.

The roles of glycan-binding proteins such as selectins and glycosylated proteins including ICAM-1 in leukocyte recruitment and transmigration have been well-documented. However, advances in experimental procedures, such as glycomic profiling now enable researches to investigate glycans and the glycomic profiles in cells and tissues in much more depth. Nevertheless, the research in this field is still in its infancy and requires further exploration to uncover the potential use of glycans as significant therapeutic targets. This review will provide an overview over glycans and novel glycan-binding proteins in leukocyte trafficking and how they are regulated. Finally, we will discuss how glycans and glycan-binding proteins can be targeted by therapeutics using glycan analogues, antibodies, lectins and glycomimetics to treat inflammatory diseases.

## Glycans and Glycan Binding Proteins in Leukocyte Capture and Rolling

**Figure 3:**
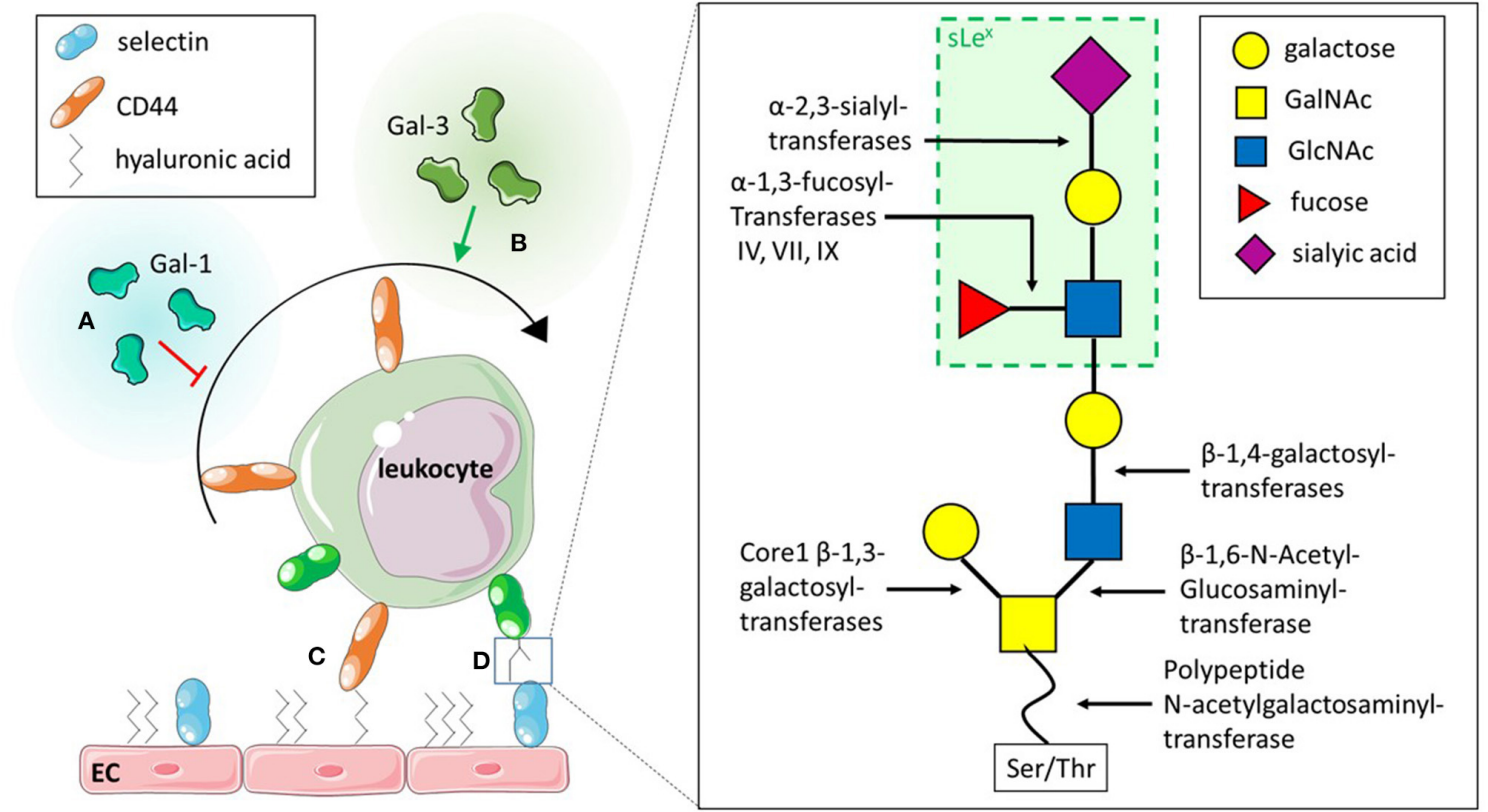
Role of glycans and glycan binding proteins in leukocyte capture and rolling. **(A)** Exogenous Galectin (Gal)-1 inhibits capture and rolling of leukocytes. **(B)** Exogenous Gal-3 on the other hand promotes capture of leukocytes. **(C)** CD44 on leukocytes and the glycosaminoglycan hyaluronic acid on the endothelial cell (EC) surface interact to contribute to leukocyte capture. **(D)** Selectins are glycan binding proteins which bind to specific O-glycan structures such as sLe^x^. These glycan structures are formed through a multitude of enzymes which catalyse the addition of different carbohydrates to the glycan precursor.

The initial interactions between the endothelium and leukocytes is mediated by selectins, a group of C-type lectins. Three types of selectins have been identified to date in mammals: (I) E-selectin, which requires transcriptional activation and is expressed on the endothelium upon pro- inflammatory stimulation with e.g. tumour necrosis factor (TNF) a (Gedeit, 1996); (II) L-selectin, expressed on leukocytes, but shed upon activation with e.g., N-Formylmethionyl-leucyl-phenylalanine (fMLP); and (III) P-selectin, expressed by platelets and endothelial cells, where it is stored in granules and mobilised to the cell surface upon activation of the endothelium. These selectins all bind to core 2 O-glycans on glycosylated proteins such as PSGL-1 and CD44. E- and P-selectins bind to glycans on leukocytes whereas L-selectin can bind core 2 O-glycans on endothelium and leukocytes. The generation of these core 2 O-glycans is facilitated by a range of different glycosyltransferases (Figure 3D). Core 1 ß-1,3-galacotsyltransferase, an enzyme catalysing the transfer of galactose from UDP-alpha-D-galactose to a beta-N-acetylgalactosamine, forms the core 1 backbone which is the basis of core 2. ß-1,6-N-acetylglucosaminyltransferase-1, ß-1,4-galactosyltransferases, α-2,3-sialyltransferases and α-1,3-fucosyltransferases, enzymes catalysing the transfers of N-acetylglucosamine, galactose, sialic acid and fucose respectively, further extend core 2 O-glycans (Figure 3D) (Sperandio et al., 2006; Buffone et al., 2013; Mondal et al., 2013; Wright and Cooper, 2014) and have been demonstrated to affect leukocyte recruitment *in vivo* (Weninger et al., 2000; Sperandio et al., 2006). For example, it was shown that leukocytes from mice with genetic ablation for both α-1,3-fucosyltransferase (Fut) IV and VII resulted in significant inhibition of rolling as observed using intravital microscopy of the post-capillary and collecting venules of mice ears (Table 1). The authors also found that rolling velocities were significantly increased in single knockouts for either Fut IV or VII (Weninger et al., 2000). Studies using bone marrow derived neutrophils from α-1,3-fucosyltransferase IV, VII and IX deficient mice (*Fut4*^−/−^, *Fut7*^−/−^, and *Fut9*^−/−^ respectively) as well as corresponding knock downs in human cell lines further confirmed the importance of fucosylation of PSGL-1 in leukocyte rolling. The knock down of *Fut7* and to a lesser extent *Fut4* and *Fut9* in human leukocytic HL-60 cells as well as in murine bone-marrow derived neutrophils decreased leukocyte interactions with recombinant selectins under hydrodynamic shear stress (Buffone et al., 2013). Polypeptide N-acetylgalactosamine transferase-1 (ppGalNAcT-1), which links the glycan molecule to the peptide (Figure 3D), has been also shown to play a crucial role in glycosylation of ligands for P-selectin (Tenno et al., 2007). More recently, its role in leukocyte rolling, adhesion and transmigration *in vivo* was characterised. These steps in the leukocyte trafficking cascade were significantly impeded in TNFα-treated cremaster muscles of ppGalNacT-1 knock out (*Galnt1*^−/−^) mice compared to littermate controls (Table 1). Chimera experiments suggest that the presence of the enzyme in hematopoietic cells is crucial for recruitment since less neutrophils migrated to the peritoneum after i. p. injection of thioglycollate in the *Galnt1*^−/−^ mice who received bone marrow from *Galnt1*^−/−^ animals compared to *Galnt1*^−/−^ mice who received bone marrow from *Galnt*^+/+^ mice (Block et al., 2012). While these studies highlight the importance of various enzymes in leukocyte recruitment and their expression in certain cell types, other studies have shown that the glycosylation of cells can depend on their state. For example, naïve T-cells do not synthesise core 2 O-glycans and therefore do not bind to P-and E-selectins. Only upon stimulation T-cells increase the expression of enzymes encoded by *Gcnt1* and *Fut7*, which generate core 2 glycans and enable activated T-cells to bind selectins (Buffone et al., 2013; Chen et al., 2016; Hobbs and Nolz, 2017). Not just glycans are altered, core 2 bearing glycoprotein CD43 has been found to be upregulated on activated T-cells, providing increased binding sites for E-selectin and therefore enabling capture and migration (Matsumoto et al., 2005, 2007; Fuhlbrigge et al., 2006; Alcaide et al., 2007; Clark and Baum, 2012). Lymphocytes are not the only cells to alter their glycosylation upon stimulation. Other studies have shown that the glycosylation changes upon stimulation also occur in monocytes: for example, PSGL-1 and sialyl Lewis X (sLe^x^) have both been demonstrated to be upregulated on human CD14^+^ monocytes in a time-dependent manner upon IL-1ß stimulation (Kanabar et al., 2016). Interestingly, the same study showed that the ß-1,4-galactosyltransferase inhibitor, 5-(5-formylthien-2-yl) UDP-Gal, could prevent IL-1ß-mediated increase in PSGL-1 and sLe^x^ without affecting basal levels of the protein and sugar (Kanabar et al., 2016). However, the study did not address the effects of IL-1ß-mediated upregulation of PSGL-1 or the impact of treatment with the inhibitor on monocyte trafficking. Nevertheless, it highlights the therapeutic potential of targeting glycosyltransferases, especially, since the basal levels of PSGL-1 and sLe^x^ remained unaffected by the treatment. Other studies have also targeted glycosyltransferases acting in the generation of sLe^x^ or sialyl Lewis a (sLe^a^), another core 2 glycan recognised by selectins (Rillahan et al., 2012). Even though inhibitors were able to interfere with the activity of the enzymes, their application *in vivo* remains limited due to off-target effects such as renal injury and difficulties in the delivery to the target site (Galeano et al., 2007; Patel et al., 2017). Interestingly, a recent study by May et al. (2020) has shown that alternative splicing of PGANTs, the *Drosophila* analogues of mammalian ppGalNTs (Table 1), which catalyse the addition of the glycan to serine or threonine, can alter the substrate and peptide preference of the enzyme. Even though this study investigates *Drosophila* PGANTs, a previous study has demonstrated the presence of splice variants in humans (Festari et al., 2017). Whether the splice variants of human ppGalNTs also affect the recognition of substrate in the same manner as the *Drosophila* splice variants and whether this impacts leukocyte recruitment remains unknown. Nevertheless, these findings offer a novel insight into previously unknown regulatory mechanisms of these enzymes which could be targeted by drugs. By targeting a more specific splice variant rather than all variants of one enzyme, it may offer a more precise treatment with less off-target effects.

**Table 1:** Enzymes involved in the capture of leukocytes.

**Enzyme type**		**Enzmye function**	**Effect on Leukocyte Capture**	**References**
Polypeptide N-acetylgalactosmaine transferase	ppGalNT1	Connects glycan to peptide	Crucial in glycosylation of ligands for P-selectin Knock down impedes leukocyte rolling, adhesion and transmigration Drosophila analogue of mammalian ppGalNT, alternative splicing alters substrate and peptide preference	Tenno et al., 2007 Block et al., 2012 May et al., 2020
	PGANT			
Fucosyltransferase	Fut IV Fut VII Fut IX	Addition of fucose to glycan	Necessary for fucosylation of PSGL-1 Knock downs decrease interaction with selectins *in vitro* and *in vivo* under flow Expression of Fut VII increased in activated T-cells so they can bind to selectins	Buffone et al., 2013 Chen et al., 2016 Hobbs and Nolz, 2017

Not only selectin-glycan interactions mediate leukocyte capture and rolling: the interaction between CD44 and the glycosaminoglycan (GAG) hyaluronic acid (HA) (Figure 3C) has previously been described to contribute to lymphocyte rolling *in vitro* (DeGrendele et al., 1996) and C-AM labelled leukocyte rolling *in vivo* (Xu et al., 2002). Further roles of the interaction between CD44 and HA in leukocyte trafficking have been reviewed elsewhere (McDonald and Kubes, 2015).

Although the changes in glycosylation of cells evidently contribute to the regulation of leukocyte rolling, other modes of regulation have been described. Galectins, a family of ß-galactoside binding proteins have been demonstrated to affect leukocyte rolling. Interestingly, even though from the same protein family, different galectins have been shown to affect leukocyte migration in opposing manner. Exogenous Gal-1, for example was described to inhibit rolling of polymorphonuclear cells (PMN) *in vitro* as well as *in vivo* during acute inflammation (Figure 3A) (La et al., 2003; Cooper et al., 2008). Conversely, endogenous chimera-type Gal-3 has been reported to promote recruitment of PMN and lymphocytes *in vivo* (Alves et al., 2013; Gittens et al., 2017). Impaired slow rolling and emigration was observed in *Gal3*^−/−^ mice during acute inflammation, while the administration of recombinant Gal-3 reduced rolling velocity and increased the number of adherent neutrophils and monocytes *in vivo* (Gittens et al., 2017) (Figure 3B). The *in vitro* models support direct effects of Gal-1 and−3 on leukocyte migration (Figures 3A,B). However, *in vivo* studies using endothelial-specific knock out mice or bone marrow chimera models could help to distinguish between the role of endogenous galectins in hematopoietic and non-hematopoietic cells in context of recruitment (Suryawanshi et al., 2013; Robinson et al., 2019). Interestingly, various studies have demonstrated increased levels of soluble galectins in serum or plasma of patients with inflammatory diseases such systemic sclerosis, atherosclerotic stroke and systemic lupus erythematosus (He et al., 2017; Chihara et al., 2018; Matsuoka et al., 2020). Whether these increased levels of soluble protein affect the migration of leukocytes in these inflammatory diseases remains unknown, but merit further investigation.

## Glycans and Glycan Binding Proteins in Leukocyte Adhesion

**Figure 4:**
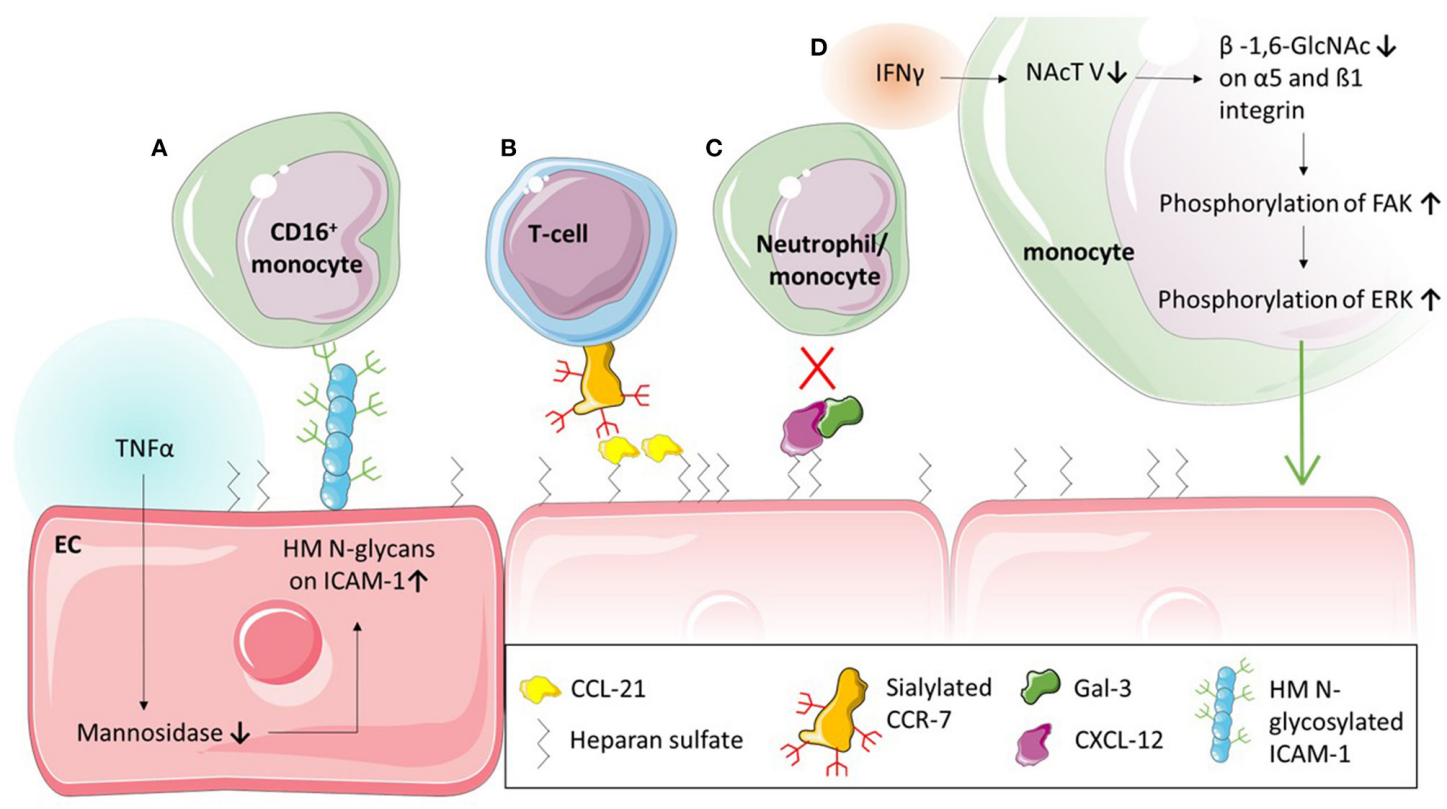
Glycans and glycan binding proteins in leukocyte adhesion. **(A)** Tumour necrosis factor (TNF) α stimulation of endothelial cells (EC) leads to a decrease of mannosidases and in turn to an upregulation of high mannose (HM) ICAM-1 on the EC. This glycoform of ICAM-1 selectively causes the adhesion of CD16^+^ monocytes. **(B)** Chemokines such as CCL-21 are bound to the endothelial surface via glycosaminoglycan heparan sulfate and interact with sialylated CCR-7 to mediate T-cell adhesion. **(C)** Glycan binding protein Galectin (Gal)-3 forms heterodimers with CXCL-12 which inhibit adhesion of neutrophils and monocytes *in vivo*. **(D)** Interferon (IFN) γ stimulation of monocytes reduces N-acetylglucosaminyltransferase (NAcT) V, which in turn decreases glycosylation of α5 and β1 integrin. This increases the phosphorylation of Focal adhesion kinase (FAK) and consequently Extracellular signal-regulated kinase (ERK), resulting in increased adhesion of monocytes.

While the interaction between PSGL-1 and selectins is critical in the first stage of leukocyte recruitment, integrins such as Mac-1 and LFA-1, and adhesion molecules such as ICAM-1 and VCAM-1, are known to facilitate firm adhesion. Nevertheless, these two steps in the recruitment cascade must not be seen as separate entities: the interaction between PSGL-1 and selectins is required for E-selectin-mediated direct activation of integrins (Taylor et al., 1996; Schaff et al., 2008; Chase et al., 2012; Morikis et al., 2017) or for the exposure of rolling leukocytes to chemokines on the endothelium which triggers G-protein coupled receptors (GPCR)-mediated integrin activation (Tsang et al., 1997; Stadtmann et al., 2013) on leukocytes followed by firm adhesion. The interaction between O-glycans and L-selectin has been demonstrated to be vital in regulating integrin activation and thereby mediating neutrophil adhesion *in vitro* and *in vivo* (Stadtmann et al., 2013). Recently, several studies have also highlighted the role of glycans on Mac-1, an integrin made up of an alpha subunit (αM; CD11b) and beta subunit (β2; CD18), in neutrophil adhesion and migration (Zen et al., 2007; Brazil et al., 2016; Saggu et al., 2018; Kelm et al., 2020). Saggu et al. (2018) showed that the CD18 subunit of Mac-1 interacts with FcγRIIA to reduce the affinity to IgG and therefore inhibit FcγRIIA-mediated neutrophil recruitment. They further demonstrated that this interaction was glycan dependent and mediated between the αI-domain of CD18 and sialylated glycans on FcγRIIA. The glycosylation of integrins is also important for adhesion of monocytes (Yang et al., 2012). Yang et al. (2012) demonstrated that the pro-inflammatory cytokine interferon (IFN)γ changes the glycosylation of monocytes which affects their adhesion and migration (Figure 4D). More specifically, they found that the treatment with IFNγ downregulated N-acetylglucosaminyltransferase V which affected the levels of ß-1,6- linked GlcNAc on integrins α5 and ß1 without affecting their protein levels (Table 2). The decrease in N-acetylglucosaminyltransferase V increased the phosphorylation of focal adhesion kinase, which in turn phosphorylates Extracellular Signal-regulated Kinase (ERK). Utilising an ERK inhibitor, they were able to inhibit the IFNγ-mediated monocyte adhesion and transmigration (Yang et al., 2012).

**Table 2:** Enzymes involved in Leukocyte Adhesion and Transmigration.

**Enzyme type**		**Enzyme Function**	**Effect on Leukocyte adhesion and transmigration**	**References**
N-acetylglucosaminyl-transferase	GNT-V	Addition of GlcNAc to OH-group of α-linked mannose	IFNγ-mediated decrease of GNT-V caused decrease of β-1,6-linked GalNAc on integrins, resulting in IFNγ-mediated adhesion of monocytes	Yang et al., 2012
Mannosidase		Remove mannose from glycan	TNFα treatment of EC downregulates mannosidase in turn resulting in high-mannose N-glycans on EC High-mannose ICAM-1 selectively recruits non-classical (CD16^+^) monocytes Oscillatory shear stress increases mannose on EC	Chacko et al., 2011 McDonald et al., 2020 Scott et al., 2012
Sialyltransferase	St6Gal1	Addition of α-2,6-linked sialic acid to terminal N-glycans	Needed for PECAM-1 presentation on EC surface	Kitazume et al., 2010

Evidently, the correct glycosylation of leukocytic proteins contributes greatly to the regulation of leukocyte migration. However, in the adhesion process, glycoproteins on the endothelium such as ICAM-1 and VCAM-1 also play a crucial role (Ley et al., 2007). They are expressed by the endothelium upon inflammatory stimuli and interact with integrins such as Mac-1 and LFA-1 (Diamond et al., 1991; Diamond and Springer, 1993). The importance of glycosylation in this interaction has been explored in a series of studies (Diamond et al., 1991; Sriramarao et al., 1993). Interestingly, contrary to O-glycan-mediated capture and rolling, N-glycans are the main type of glycans involved in adhesion. Particularly one type of N-glycan, high-mannose N-glycans (Figure 1B), increase monocyte adhesion (Figure 4A) (Scott et al., 2012). Specifically, high-mannose glycans on ICAM-1 have been demonstrated to drive the adhesion of the human monocytic cell line THP-1, independent of ICAM-1 and E-selectin expression (Chacko et al., 2011), as well as rolling and adhesion of primary monocytes (Scott et al., 2013a,b). This appears to be a mechanism occurring during acute inflammation; the pro-inflammatory cytokine TNFα, for example has been shown to downregulate mannosidases, enzymes which remove mannoses from glycans, due to hydrogen peroxidase released from endoplasmatic reticulum oxidoreductase-1-α (Figure 4A) (McDonald et al., 2020). This TNFα-mediated reduction in mannosidases in turn results in an increase of high-mannose N-glycans on the endothelial surface (Table 2) (Chacko et al., 2011; McDonald et al., 2020). Whether this is also occurring in chronic inflammation remains unknown. However, not only inflammatory cytokines appear to increase high mannose N-glycan levels on endothelium: oscillatory shear stress has been shown to increase these types of glycans too (Table 2) (Scott et al., 2012). Subsequent studies have shown that the increase of ICAM-1 glycosylated with mannose-rich glycans (high mannose ICAM-1) has functional consequences. High mannose ICAM-1 selectively recruits non-classical/intermediate (CD16^+^) monocytes over classical (CD16^−^) monocytes in a Mac-1 dependent manner (Regal-McDonald et al., 2019). Interestingly, studies using human and murine samples have shown that this high-mannose ICAM-1 glycoform is present in atherosclerotic lesions (Scott et al., 2012; Regal-McDonald et al., 2020) and positively correlates with macrophage burden in these lesions. α-2,6-sialylated ICAM-1 levels on the other hand, did not associate with increased macrophage content in lesions (Regal-McDonald et al., 2020). However, whether the selective recruitment of CD16^+^ monocytes by high-mannose ICAM-1 contributed to increased macrophage content in high-mannose positive atherosclerotic lesions still remains to be determined. The selectivity of recruitment caused by differential glycosylation of adhesion proteins is an interesting area for drug targeting. A study by Chacko et al. (2011) showed that the PPARγ agonist rosiglitazone, an anti-diabetic drug, reduced TNFα-induced expression of high-mannose N-glycans and successfully reduced adhesion of THP-1 cells and primary human monocytes under physiological flow. Whether this PPARγ agonist can also prevent increases in high-mannose glycans caused by oscillatory shear stress have not been established. While rosiglitazone successfully reduced the adhesion of monocytes, it has also been linked to an increased risk of cardiovascular disease (Chen et al., 2012) and therefore may not be a suitable therapeutic option. Other *in vitro* studies targeting high mannose glycans with specific antibodies or lectins have successfully decreased monocyte adhesion further highlighting the therapeutic potential of targeting these glycan structures (Scott et al., 2012), however, these effects need to be validated *in vivo*.

Chemokine-mediated arrest and spreading of leukocytes has been shown to also depend on GAGs such as heparan sulphate (Middleton et al., 2002). More recently, various studies showed that chemokine presentation such as that of CXCL-8 and CCL-21 depend on GAGs (Figure 4B) (Proudfoot et al., 2003; Bao et al., 2010; Weber et al., 2013; Joseph et al., 2015; Goldblatt et al., 2019). Bao et al. (2010) demonstrated that mutant heparan sulphate leads to diminished chemokine presentation, resulting in decreased integrin-mediated recruitment of lymphocytes *in vivo* while specifically CCL-21 has been demonstrated to be immobilised by GAGs on the cell surface (Weber et al., 2013). Furthermore, the ablation of GAGs on neutrophils *in vitro* resulted in reduced chemotaxis towards CXCL-8 (Goldblatt et al., 2019). These studies suggest that the immobilisation of chemokines through GAGs on the cell surface contribute to a high local concentrations of chemokines, concentration gradients and chemokine-receptor binding, all potentially modulating leukocyte migration. However, more studies are required to understand the regulatory mechanisms involved in GAG-chemokine interactions and their effect on leukocyte migration. Particularly tissue-specific differences in these interactions, as suggested by Gangavarapu et al. and others (Gangavarapu et al., 2012; Rajarathnam et al., 2018), are of interest in order to understand tissue-specific differences in leukocyte trafficking. A range of studies have also highlighted the importance of glycosylation of chemokines (Ludwig et al., 2000; Frommhold et al., 2008). Glycosylation, especially sialylation, was found to contribute to chemokine binding to their cognate receptors (Frommhold et al., 2008; White et al., 2013; Doring et al., 2014; Su et al., 2014; Wright and Cooper, 2014; Hauser et al., 2016). For example, sialyation of CCR-7, a receptor of CCL-19 and−21, inhibits its signalling and therefore migration of T-cells *in vitro* (Figure 4B) (Hauser et al., 2016). Interestingly, dendritic cells release enzymes which can de-sialylate CCR-7 and therefore increase T-cell chemotaxis (Hauser et al., 2016). However, not only chemokine-chemokine receptor interactions are dependent on glycosylation. More recently, a study revealed how glycan-binding protein Gal-3 can mediate chemokine function. The study revealed Gal-3 forms heterodimers with CXCL12 (Figure 4C) (Eckardt et al., 2020), a chemokine known to interact with CXCR4. This interaction between CXCL12 and CXCR4 is known to modulate tissue infiltration of neutrophils and monocytes in myocardial infarction and atherosclerosis (Zernecke et al., 2008; Liehn et al., 2011; De Filippo and Rankin, 2018). Eckardt et al. (2020) showed that Gal-3 inhibited CXCL12 mediated migration of neutrophils and monocytes *in vitro* as well as the infiltration of the peritoneum *in vivo* (Figure 4C). The study also showed that the recruitment of classical monocytes *in vivo* was significantly increased to the peritoneum of Gal-3^−/−^ mice after thioglycollate treatment compared to wild type mice, further indicating a role for Gal-3 in CXCL12 mediated recruitment of classical monocytes. Whether circulating Gal-3, which is upregulated in various inflammatory pathologies (He et al., 2017; Dong et al., 2018; Di Gregoli et al., 2020) is also able to interfere with CXCL12 mediated leukocyte recruitment *in situ* remains unknown. The authors of the study nevertheless suggest that, based on their data, the CRD of Gal-3 may be a promising anti-inflammatory target. Other galectins have also been shown to modulate leukocyte adhesion (La et al., 2003; Norling et al., 2008; Yamamoto et al., 2008; Gittens et al., 2017); multiple studies have found that Gal-1 inhibits leukocyte extravasation (La et al., 2003; Norling et al., 2008; Iqbal et al., 2011). Conversely, several other galectins have been shown to promote leukocyte adhesion to the endothelium (Yamamoto et al., 2008). Yamamoto et al. (2008) treated peripheral blood leukocytes with Gal-8, and found increased adhesion to HUVECs which they believed was α4-integrin-dependent. An important caveat of this study was that these assays were performed under static conditions. Due to the lack of physiological flow, the leukocytes automatically come into contact with the endothelium and the effect of these galectins on the capture of leukocytes by the endothelium cannot be assessed. The use of physiological flow would help to uncover whether galectins also affect the capture, and therefore adhesion and transmigration.

These studies demonstrate the importance of glycosylation for leukocyte adhesion and suggest possible mechanisms which could be targeted by therapeutics.

## Glycans and Glycan Binding Proteins in Leukocyte Transmigration

During the final stages of the migration cascade, leukocytes transmigrate across the endothelium. This can happen in either a transcellular or paracellular manner. Similar to the previous steps of the recruitment cascade, leukocyte transmigration is regulated by a range of different proteins: including PECAM-1, JAM-A, ICAM-2 and CD99 on the endothelium.

JAM-A is known to contribute to the barrier function of the endothelium since it is located within tight junctions. It contributes to cell migration by acting as ligand for LFA-1 on leukocytes. Whether glycans play a role in this interaction remains unknown. However, a recent study has shown that a particular N-glycan at position N185 of JAM-A contributes to barrier function. The study showed that mutating the glycosylation site of JAM-A in CHO cells resulted in a decrease of LFA-1 dependent adhesion of leukocytes compared to cells expressing wild type JAM-A (Scott et al., 2015).

PECAM-1 is another key junctional molecule involved in transmigration. It forms homophilic interactions which play a key role in vascular permeability (Ferrero et al., 1995; Privratsky et al., 2011), detecting flow (Osawa et al., 2002; Tzima et al., 2005) and leukocyte transmigration (Muller et al., 1993; Nourshargh et al., 2006). Levels of PECAM-1 are reduced in sialyltransferase knock out (*ST6Gal1*^−/−^) mice (Kitazume et al., 2010). This suggests that the siaylation of PECAM-1 contributes to its presentation on the endothelial cell surface (Table 2). However, how this potentially contributes to the regulation of leukocyte migration remains unknown. A more recent study suggests that glycosylation plays a role in the homophilic interactions of PECAM-1 therefore potentially affecting leukocyte transmigration. It was found that glycans at the asparagine at position 25, which is located within the trans-homophilic binding interface of PECAM-1 contribute to the homophilic interactions (Lertkiatmongkol et al., 2016). The same study suggests that negatively charged 2,3-sialic acid moieties form electrostatic bridges with a positively charged lysine at position 89 (Lertkiatmongkol et al., 2016) which has previously been described to play a role in the homophilic interactions of PECAM-1 (Newton et al., 1997). Interestingly, 2,6 sialic acid moieties blocked the homophilic interactions. A N25Q mutant of PECAM-1, lacking the glycan at position 25 was shown to localise in the same manner as the native protein, however, the recovery of its barrier function was significantly damaged, highlighting an important role of the glycosylation in the permeability of the endothelium. How the lack of this glycan and therefore the homophilic interaction affects leukocyte migration remains unknown. Interestingly murine PECAM-1 lacks the asparagine at position 25 and therefore also the glycosylation. It has however two more glycosylation sites, a total of nine, compared to human PECAM-1 and highlights key differences of glycosylation between species. Also, how or if the glycosylation sites of PECAM-1 are affected in pathological conditions, especially during inflammation remains to be determined.

As previously mentioned, the various steps in the recruitment cascade are not separate mechanisms. Therefore, it is no surprise that glycans associated with selectins and therefore capture and rolling, can also act in transmigration, as demonstrated by various studies. The use of antibodies targeting sLe^x^ as well as the removal of sLe^x^ on Mac-1 disrupted the interaction between Mac-1 and E-selectin while causing degranulation of neutrophil secondary granules without stimulation with chemoattractants. Neutrophil transmigration across intestinal epithelial cell monolayers was also significantly decreased when the neutrophils were treated with these anti-sLe^x^ antibodies compared to neutrophils treated with a control antibody (Zen et al., 2007). A study by Brazil et al. (2016) also showed a role of Le^x^ in neutrophil transmigration when targeting with antibodies. They observed that terminal glycans rather than subterminal Le^x^ drive an increase in degranulation and a decrease in transepithelial migration of neutrophils, important in mucosa-lined organs such as the intestine and lung. Antibodies are not the only modalities used to target glycan moieties, Kelm et al. (2020) utilised lectins specifically targeting high-mannose or bi-antennary galactosylated N-glycans on CD11b and inhibited transepithelial migration among other inflammatory functions of neutrophils. Contrary to targeting Le^x^, targeting Le^a^, a stereoisomer of Le^x^, with antibodies or lectins increased transepithelial migration of neutrophils and therefore suggests a potential role of Le^a^ in the inhibition of transepithelial migration. Interestingly, the same group had previously shown that targeting the sialylated version of Le^a^, sLe^a^, on CD44v6 expressed by epithelial cells prevented transepithelial migration of neutrophils (Brazil et al., 2010, 2013). Whether the opposing results of antibodies and lectins targeting Le^x^ and Le^a^ are due to the presence of Le^x^ and Le^a^ on different proteins and therefore affecting the migration differently seems likely, since the expression of Le^x^ and Mac-1 is increased with activation of neutrophils whereas Le^a^ was not. Additionally, the ligation of Le^x^ affected multiple cellular functions including migration and apoptosis, whereas targeting Le^a^ only affected transepithelial migration (Brazil et al., 2017). Collectively, these studies highlight the potential of glycan specific antibodies and lectins as inhibitors of glycan-mediated functions.

## Inhibition of Glycan Biosynthesis or Recognition

**Figure 5:**
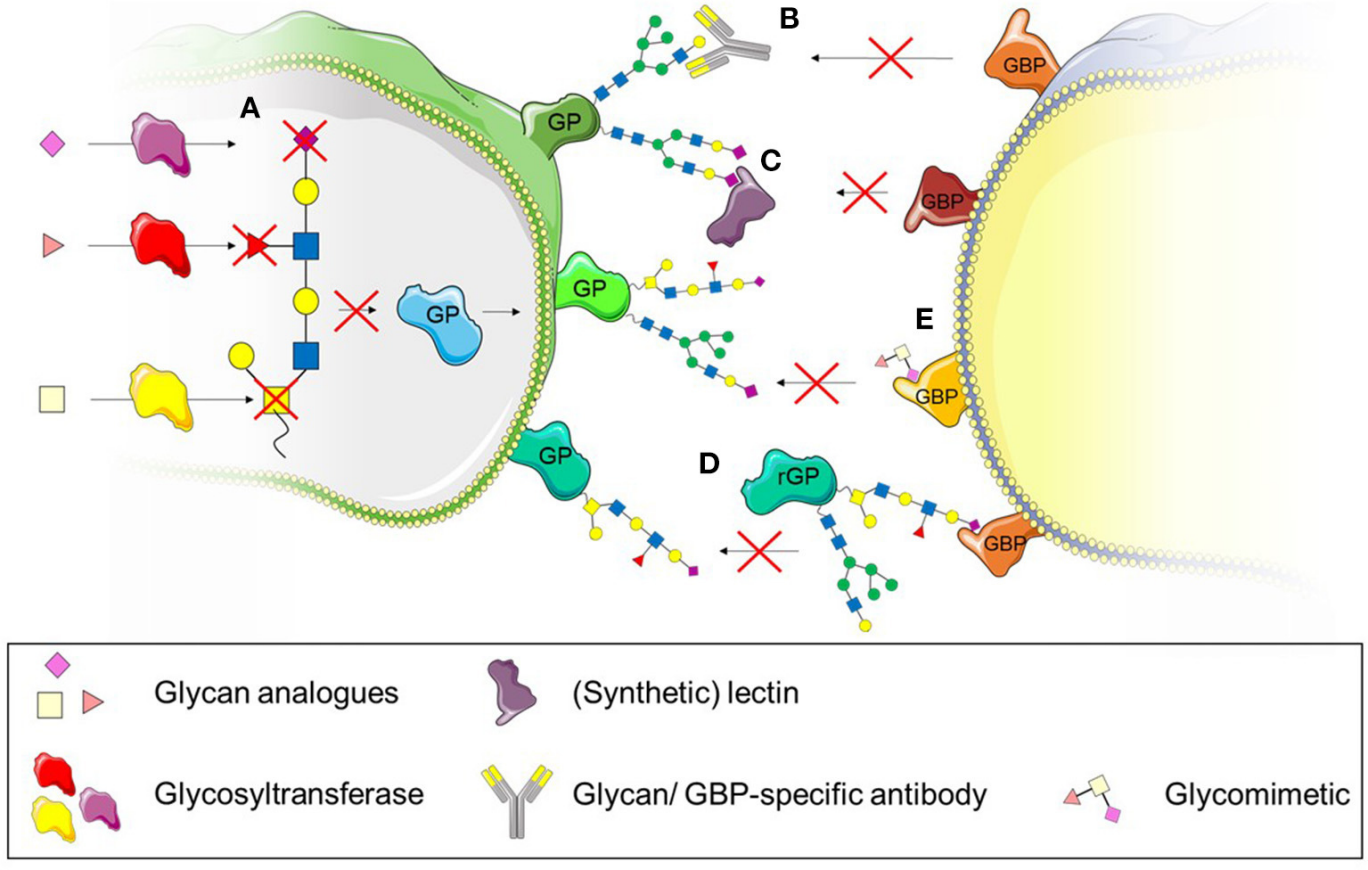
Therapeutic strategies to interfere with leukocyte recruitment and transmigration. **(A)** The use of synthetic glycan substrates impairs enzyme-catalysed glycan formation and prevents recognition by glycan-binding proteins (GBP). **(B)** Specific antibodies targeting glycans can also be used to interfere with glycan recognition by GBPs. **(C)** (Synthetic) lectins bind to specific glycan structures and interfere with recognition by GBPs. **(D)** Recombinant glycoproteins (rGP) bind to GPBs and therefore prevent binding to native glycoproteins (GP). **(E)** Glycomimetics can also be used to bind to GBPs and prevent binding to glycoproteins.

Several studies have highlighted the potential of glycosylation as a suitable therapeutic target to treat inflammatory diseases characterised by dysregulated leukocyte trafficking (Mertens et al., 2006; Gaber et al., 2011; Dwivedi et al., 2018; Kelm et al., 2020). This might be particularly promising since changes in the glycomic profile of cells and tissues have been previously described in various inflammatory diseases such as rheumatoid arthritis, ulcerative colitis and systemic lupus erythematosus (Axford et al., 1992; Gornik and Lauc, 2008; Larsson et al., 2011). Various approaches to target glycosylation can be taken: (i) interference with the biosynthesis of glycans using inhibitors of glycosyltransferases or (ii) interference with the recognition of glycans either by using targeted antibodies or lectins (Kelm et al., 2020) or (iii) blocking the glycan receptor with recombinant glycoproteins or glycomimetics (Dwivedi et al., 2018). The use of these different approaches as therapeutics to treat dysregulated leukocyte trafficking in inflammatory disease will be discussed in the following section (Figure 5).

### Interfering With Biosynthesis of Glycans–Glycosyltransferase Inhibitors

The biosynthesis of glycans is a tightly controlled pathway whereby a multitude of different glycosyltransferases are involved to generate thousands of glycan structures found in cells. Studies have revealed that the glycosylation changes in diseases (Axford et al., 1992; Gornik and Lauc, 2008; Larsson et al., 2011) and therefore, the enzymes involved in these processes may provide a suitable target. Introduction of synthetic glycan building blocks offer the opportunity to interfere with glycan recognition without disrupting enzyme activity by targeted delivery to the affected cell type or tissue. Disrupting the enzyme function in other tissues could lead to off target effects. Numerous studies have investigated the potential of such analogues i*n vitro* (Barthel et al., 2011; Rillahan et al., 2012; Agarwal et al., 2013; Jiang et al., 2016; Kanabar et al., 2016; Dwivedi et al., 2018; Moons et al., 2019) and *in vivo* (Dimitroff et al., 2003; Gainers et al., 2007; Marathe et al., 2010) (Figure 5A). These approaches targeted various glycosyltransferases such as ß-1,4-galactosyltransferase, fucosyltransferase III, V, VI and VII as well as sialyl transferases (Burkart et al., 2000) and reported reductions in the respective O- and N-glycan structures. Particularly successful were fluorinated glycan analogues. Rillahan et al. (2012) found that the fluorinated fucose analogue GDP-2F-Fuc and sialic acid analogue 3F-Ax-NeuAc inhibited various fucosyltransferases and sialyl-transferases which resulted in reduced fucosylation and sialylation of N- and O-glycans. Particularly sLe^x^ was reduced, which impaired the binding of HL-60 cells to recombinant E and P -selectin under flow.

Even though these studies demonstrate the potential of glycan analogues as inhibitors of glycan biosynthesis, there is a lack of studies investigating their effects on leukocyte migration, particularly *in vivo*. Only such studies can evaluate the true potential of these inhibitors as drugs since targeting the biosynthesis of glycans may prove challenging since glycans are implicated in virtually all cell types and tissues. Currently, too little is known about their regulation, especially in pathological conditions to make reliable predictions. And while the inhibitors and analogues may provide the necessary insight into pathways of biosynthesis as well as cellular mechanisms *in vitro*, the overlapping specificities, multi-substrate specificities and structural homology of glycosyltransferases could prove too difficult to use as therapeutic targets *in vivo* (Videira et al., 2018).

### Interfering With Glycan Recognition–Antibodies, Lectins and Glycomimetics

While glycan analogues target the biosynthesis of glycans, another approach to interfere with glycan function is to inhibit their recognition by glycan-binding proteins. We have previously mentioned studies targeting glycan structures with specific antibodies (Zen et al., 2007; Brazil et al., 2016, 2017) (Figure 5B). While these studies showed successful inhibition of glycan function through the use of specific antibodies, their potential *in vivo* remains untested. Lectins have also been previously used in *in vitro* studies to inhibit the recognition of glycans (Brazil et al., 2017; Kelm et al., 2020) (Figure 5C). Similar to antibodies, they bind specific glycan structures and interfere with recognition through glycan-binding proteins. The lectins used experimentally generally stem from natural sources such as tomatoes (*Lycopersicon Esculentum* lectin, LEL), peanuts (peanut agglutinin lectin, PNA) or mussels (*Crenomytilus grayanus* lectin, CGL) and are used as tools to investigate the function and expression of glycans rather than pharmacological agents. The use of synthetic lectins may provide a more promising approach since their specificity can be higher than of naturally occurring lectins (Ferrand et al., 2009). One study has used synthetic lectins to demonstrate their specificity for glycans as cancer diagnostics tools (Bicker et al., 2012). They used an array of various synthetic lectins and various cancerous metastatic and non-metastatic cell lines and showed that the synthetic lectins differentially bind to the glycans of these cell types, distinguishing subtle differences between healthy and different pathological glycan structures (Bicker et al., 2012). This may also be helpful as diagnostic tool in inflammatory diseases but requires further investigation. Even though these synthetic lectins provide high sensitivity, no studies, to our knowledge have used them to interfere with cellular functions in a therapeutic context.

Instead of glycans, glycan binding proteins could also be targeted to interfere with the recognition of glycans. For example, recombinant glycoproteins have been used to bind to glycan binding proteins, interfering with the binding of native glycoprotein (Figure 5D). This has been tested in clinical trials in the case of PSGL-1. Recombinant PSGL-1-immunoglobulin was successfully used *in vivo* before clinical trials in patients with myocardial infarction and renal allografts (Mertens et al., 2006; Gaber et al., 2011). Unfortunately, neither of the clinical studies could demonstrate significant beneficial outcomes for patients. Whether the glycosylation of recombinant PSGL-1 reflected the glycosylation of naturally occurring PSGL-1 is not known, but may be crucial for the success of it as a therapeutic modality. The choice of cell type producing the recombinant protein is important to ensure correct glycosylation. While the use of *Escherichia coli* (*E. coli*) is a cheaper option of generating proteins, *E. coli* glycosylation is significantly different to mammalian glycosylation (Lee et al., 2013) and has to be taken into account when generating proteins for the interference of glycan recognition. Other approaches of targeting the interaction between selectins and PSGL-1 might therefore be more promising. For example the use a mimetic of the N-terminus of PSGL-1, GsnP-6, which has successfully inhibited P-selectin function *in vitro* and *in vivo* (Krishnamurthy et al., 2015). GsnP-6 interfered in the interaction between PSGL-1 and P-selectin which resulted in increased rolling velocity of human neutrophils and monocytes *in vitro*. This PSGL-1 mimetic was also able to interfere with the interaction of P-selectin and PSGL-1 *in vivo* while inhibiting early thromboinflammatory events such as platelet aggregation and platelet-leukocyte interactions (Krishnamurthy et al., 2015). This interference may not only affect leukocyte recruitment, but may also be beneficial in inhibiting dendritic cell driven atherogenesis mediated through the interaction of P-selectin with PSGL-1 (Ye et al., 2019).

The use of glycomimetics (Figure 5E), rather than recombinant glycosylated proteins might improve the outcome of therapies targeting glycan recognition due to their high affinity to respective glycan-binding proteins and improved pharmacokinetics compared to their naturally occurring counterparts (Hevey, 2019). Synthetically generated glycomimetics can act as small molecule inhibitors by binding to glycan binding proteins with higher affinity. For example, Rivipansel (or GMI-1070), a pan-selectin targeting glycomimetic was successfully used to reduce sickle red blood cell-leukocyte interactions *in vivo* and therefore improved blood flow and survival of sickle cell mice (Chang et al., 2010). However, the Phase III, double-blind, placebo controlled clinical trial to treat vaso-occlusive events in humans with Sickle Cell Disease (clinicaltrials.gov, NCT02187003) did not meet the primary and secondary efficacy endpoints and was therefore unsuccessful^1^.

Various studies have used glycomimetics to target galectins (Figure 5D). While some glycomimetics have specific affinities to just one carbohydrate recognition domain of tandem-repeat galectins, other glycomimetics can bind to several galectins, revealing the complexity of these compounds (Pal et al., 2018, 2019; Stegmayr et al., 2019; Mahanti et al., 2020). For example, a quinolone-derivatised galactoside bound selectively to the N-terminal domain of Gal-8 (Pal et al., 2018) while 3 N-aryl galactosides were shown to selectively bind the C-terminal domain of Gal-9 and N-aryl gulosides to the N-termainal domain of Gal-9 (Mahanti et al., 2020). Their efficacy was demonstrated for extra- and intracellular galectins and they were able to interfere with cellular functions dependent on the respective galectin (Delaine et al., 2016; Stegmayr et al., 2019). Glycomimetics targeting Gal-3 have been shown to block extracellular binding of Gal-3 to CHO cells and also successful in inhibiting intracellular accumulation of Gal-3 around the disrupted membrane of intracellular vesicles of JIMT-1 breast cancer cells while having a low basal toxicity (Stegmayr et al., 2019). The inhibitor GB0139 (formerly TD139) targeting Gal-3 has been tested as therapeutic in lung fibrosis, successfully passed Ib/IIa clinical trials, and has moved into IIb trials which are currently in progress (clinicaltrials.gov; NCT03832946). Another glycomimetic Gal-3 inhibitor (Salameh et al., 2010), Cpd47, blocked Gal-3 inhibition of insulin-stimulated glucose transport in L6 myocytes and has also been tested in Type 2 diabetes models *in vivo*. The Gal-3 inhibitor improved the glucose tolerance in obese mice after a single dose of the inhibitor as well as continuous administration over 2 weeks via a minipump, suggesting a promising treatment for Type 2 diabetes in acute and chronic settings (Li et al., 2016).

These studies clearly demonstrate the potential of glycomimetics as therapeutics. Whether they could also interfere with glycan-dependent steps of the leukocyte recruitment cascade remains to be tested.

## Discussion

Here, we have reviewed novel roles of glycans in leukocyte recruitment and transmigration as well as their potential as therapeutic targets in the treatment of inflammatory diseases. While the roles of glycans in leukocyte recruitment were reported decades ago, more recent research has uncovered roles in virtually all steps of the pathway. Each step in the recruitment cascade has a number of enzymes involved which can alter the glycosylation of certain proteins and therefore the capture, adhesion and transmigration of leukocytes. More specifically, this review has shown, that the enzymes can act in a tissue- or leukocyte subset-dependent manner. However, differences between acute and chronic inflammatory settings, the translatability of preclinical *in vitro* and *in vivo* studies still requires more validation. Various studies have also highlighted the potential of targeting glycan biosynthesis by interfering with enzyme activity or by targeting glycan recognition directly with specific antibodies, lectins or glycomimetics. However, to be able to target these safely, more studies are needed to limit off-target effects. Nevertheless, the inflammation-dependent changes in glycosylation provide a promising therapeutic target.

## Author Contributions

FK and AI wrote the manuscript. AI edited the manuscript. All authors contributed to the article and approved the submitted version.

## Conflict of Interest

The authors declare that the research was conducted in the absence of any commercial or financial relationships that could be construed as a potential conflict of interest.

## Footnotes

^1^https://www.pfizer.com/news/press-release/press-release-detail/pfizer_announces_phase_3_top_line_results_for_rivipansel_in_patients_with_sickle_cell_disease_experiencing_a_vaso_occlusive_crisis
